# Nursing students in isolation during the COVID-19 pandemic: A phenomenological study

**DOI:** 10.4102/hsag.v30i0.2805

**Published:** 2025-01-23

**Authors:** Vistolina Nuuyoma, Frieda Makambuli

**Affiliations:** 1 School of Nursing and Public Health, Faculty of Health and Veterinary Medicine, University of Namibia, Windhoek, Namibia; 2 School of Nursing and Public Health, Faculty of Health and Veterinary Medicine, University of Namibia, Rundu, Namibia

**Keywords:** COVID-19 pandemic, coping mechanisms, isolation, resilience, nursing education, nursing students

## Abstract

**Background:**

Isolation as a public health practice encompasses physical and social separation of individuals from others, and it was key in preventing human-to-human spread of the coronavirus disease 2019 (COVID-19) virus. Yet, experiences of patients in isolation have been mostly studied in the general population and qualified health professionals, excluding nursing students who were also part of the frontliners and therefore, part of the population at risk of infections.

**Aim:**

The study explored how nursing students who tested positive for the COVID-19 virus have experienced the process of being in isolation and understanding their source of resilience.

**Setting:**

Northeastern Namibia.

**Methods:**

A qualitative phenomenological study was conducted. Data were collected from 14 nursing students via face-to-face and telephone interviews, while recruitment was conducted through purposive and snowballing sampling techniques. Data were analysed by interpretative phenomenological analysis.

**Results:**

Five themes that emerged from data analysis are the physiological spectrum, psychosocial spectrum, the value of isolation, source of resilience and coping mechanisms and the impact of isolation.

**Conclusion:**

Students experienced isolation as a period of reflecting on how their symptoms commenced and progressed from minor to major. Students’ sources of resilience and coping mechanisms were to remain opportunistic, learn new skills and lean on family support. Moreover, isolation negatively affected students’ academic life and human connectedness.

**Contribution:**

The findings have implications in preparation for future pandemics, promoting the resilience and mental health of nursing students. Moreover, they may assist in developing a coordinated counselling and psychological support system for nursing students.

## Introduction

Pneumonia cases of unknown aetiology have been confirmed in Wuhan City, Hubei Province, China, later identified as a novel coronavirus disease 2019 (COVID-19) (Lu, Stratton & Tang 2020:401). On 30 January 2020, the World Health Organization (WHO) proclaimed the virus as a global concern that requires public health emergency responses and was declared a pandemic on 11 March 2020. The virus infected approximately 760 million people and caused seven million deaths globally (WHO 2023:n.p.). Health care workers (HCWs) were reported to be among the highest population groups at risk of contracting the virus during the COVID-19 pandemic, with nurses mostly infected (Bandyopadhyay et al. 2020:4; Sabetian et al. 2021:2). Owing to the clinical practice of nursing programme that requires attachment to health care facilities and community-based settings, nursing students are considered as frontliners and, therefore, were part of the population at risk of contracting the virus.

World Health Organization recommended people who developed symptoms of the COVID-19 virus or have tested positive self-isolate until recovery and people with severe symptoms admitted in isolation wards and mild cases were recommended to be managed in community-based isolation centres (WHO 2023:n.p.). According to the Centers for Disease Control and Prevention (CDC 2023:n.p.), isolation is a public health practice used to separate patients with a contagious disease from people who are not sick, and it is usually done till individuals are no longer able to transmit the pathogen. This encompasses the physical and social separation of individuals from others. The practice of isolation differs from quarantine; the latter refers to the restrictions of movement of persons who are suspected to have been exposed to a contagious disease but are not yet ill. This is because they either did not become infected or because they are still in the incubation period (Wilder-Smith & Freedman 2020:1). People in quarantine are closely monitored to observe for appearance of any signs and symptoms following which they are transferred into isolation immediately. During the COVID-19 pandemic, isolation was practiced for 5–10 days, or sometimes till 14 days, depending on whether an individual is asymptomatic or symptomatic. This was key in curbing the skyward trend of COVID-19 infection rates because of the human-to-human spread of the virus being the mode of rising new cases (Nam et al. 2021:11). Despite this benefit, social isolation is reported to have contributed to chronic loneliness and boredom, which may have detrimental effects on the physical and mental well-being of an individual (Banerjee & Rai 2020:526). It is worth noting that isolation during the COVID-19 pandemic was associated with depression, anxiety and stress as a result of being separated from family and close friends (Liozidou et al. 2023:1267); some patients in isolation had experienced negative emotions, doubt of treatment provided and uneasiness about modifying their daily life (Pei et al. 2021:1115); while Williams et al. (2020:3) reported lack of trust in and clearness of government communication around isolation; patients in isolation observed that others did not adhere to protocols and had concerns around their future and social reintegration after isolation. Generally, there was mental health instability and moral anguish among caregivers (Mulaudzi et al. 2022:3). Notably, these studies were conducted on the general population and not nursing students.

Isolation has a negative impact on human connectedness considering that African principles of wellness and caring for sick people are rooted in the community and the human being is viewed as nothing without the community (Chigangaidze, Matanga & Katsuro 2022:322). From an African perspective, individuals believe in an *Ubuntu* philosophy, which calls for cohesion in the community and a caring attitude towards one another. It entails a welcoming attitude, which constitutes openness to sharing, to be generous, caring and compassionate (Nolte & Downing 2019:15). Therefore, *Ubuntu* was seen as a significant philosophy in fighting the COVID-19 pandemic as it supports generosity, food security, holism, spirituality, social justice and human rights, sharing and team spirits, self-awareness and social responsibility, promotion of health, personal hygiene and environmental justice (Chigangaidze et al. 2022:322). However, this was constrained by isolation practices and non-visitation policies in healthcare settings.

COVID-19 pandemic required individuals to resort to being resilient as a coping mechanism, and it is one of the constructs that enhance mental health (Skalski et al. 2022:2). Resilience refers to the ability to bounce back from difficult experiences, and it encompasses the ability to adapt well in the face of danger, tragedy, trauma or significant sources of stress (Sarra & Berman 2017:457). In addition, Mohammadi et al. (2022:2) defined resilience as the capability to acclimatise to life-threatening situations effectively. Before the COVID-19 pandemic, there have been records of how *Ubuntu* philosophy may be utilised as a tool for resilience (Sarra & Berman 2017:455). During the COVID-19 pandemic, *Ubuntu* philosophy has been a source of resilience, because of the notion of togetherness and collaboration in enduring challenging situations. Moreover, the environment or society in which an individual resides influences the ability to cope with and recover from traumatic events (Sumner et al. 2023:5). As a source of resilience, people undergoing COVID-19 treatment reported experiencing social support and having faith in the community and nation (Thompson et al. 2023:5). This has links to how the *Ubuntu* philosophy is used to promote resilience.

On the other hand, the COVID-19 pandemic required social fencing, which was practiced by prohibiting cultural, religious, mass political and educational gatherings in the community (Khongsai et al. 2021:130). This implies that the closure of educational institutions was also crucial to decreasing the incidence of new COVID-19 cases (Nam et al. 2021:12). Many nursing programmes resorted to remote learning as an option to continue theoretical teaching during the pandemic. However, nursing students were still required to attend practical sessions and clinical practice in healthcare facilities. Nursing students’ perceptions and experiences of transiting to virtual learning during the COVID-19 pandemic are widely researched and understood in different contexts (Boman et al. 2022:4; Martens et al. 2024:71; Molefe & Madunda 2022:4; Nuuyoma, Lauliso & Chihururu 2023:5; Rood, Tanzillo & Madsen 2022:3; Wallace et al. 2021:614). In addition, there are shreds of evidence of studies conducted on experiences of nursing students on caring for patients during the COVID-19 pandemic (Dempsey et al. 2023:146; Farfán-Zúñiga et al. 2022:6; Godbold et al. 2021:3; Gómez-Moreno et al. 2022:3; Mohammadi et al. 2022:4; Nabavian, Rahmani & Alipour 2021:3). These suggest evidences available on educational experiences of nursing students during the COVID-19 pandemic; however, there are evidence and empirical gaps, as well as population gaps in the available literature on experiences of nursing students as patients who tested positive for the COVID-19 virus and how they had undergone isolation. It is with this background that this study helped address these gaps by exploring how nursing students who tested positive for the COVID-19 virus have experienced the process of being in isolation and understanding their source of resilience. This may help nursing students, student counsellors, nurse educators and professionals to understand the isolation process and source of resilience for nursing students and may provide evidence to use for future pandemics and similar challenging events.

## Research methods and design

### Study design

This research followed a qualitative phenomenological approach as the study design. A phenomenological study examines humans through descriptions provided by the people who are involved in the phenomenon and therefore was chosen as it is suitable for studying lived experiences and how people interpret them (Brink, Van der Walt & Van Rensburg 2018:105). This was guided by the constructivist-interpretative paradigm. The phenomenological design helped the researcher to be engaged in the process of bracketing, intuiting, analysing and describing, which was necessary for understanding the experiences of nursing students who tested positive for COVID-19, how they have experienced the process of being in isolation and their source of resilience. Standards for reporting qualitative research (SRQR) by O’Brien et al. (2014:1247) were used as a guiding tool for improving the quality of reporting in this study.

### Setting

The study was conducted at one of the public university’s campuses located in northeastern areas of Namibia. The campus registers students to pursue academic programmes in the fields of education, economics, management sciences and nursing. For a nursing programme, a 4-year, full-time undergraduate Bachelor of Nursing Science (clinical) Honours is offered. The town where the campus is located had a high number of COVID-19 cases and had a community-based isolation centre, which is an institutional-based isolation for people who tested positive. Because of limited spaces at the community-based centre, self-isolation was allowed at patients’ places of residence under the supervision of family members and community monitoring teams. Patients requiring admission were cared for in a COVID-19 isolation centre at an intermediate hospital in town. Nursing students who tested positive for COVID-19 were required to report to the clinical practice coordinator at the campus for support, monitoring and prevention of the spread of the virus.

### Population and sampling strategy

The target population comprised nursing students registered for a degree nursing programme at a campus where the study was conducted. The campus has reported approximately 30 cases of confirmed COVID-19-positive tests among nursing students during the first phase of the pandemic in 2020. However, several students who tested positive were not reporting themselves because of delays in receiving laboratory results.

To recruit participants, researchers shared participants’ information sheets consisting of the study purpose, objectives, the process of data collection and details of the researchers. This was done through nursing students’ WhatsApp groups. After a long period of non-response, the first two participants were solicited through purposive sampling, whereby the researcher approached and invited two nursing students who were known to have tested positive for the COVID-19 virus to participate in the study. The researchers were comfortable to approach the two students as they had previously announced their positive results on WhatsApp groups as a precaution to other students. The two students agreed to participate in the study and appointments for interviews were made with respect to time, date and mode of interview. They were asked for referrals to other students who also tested positive for the COVID-19 virus. Therefore, the study was following a snowballing sampling (Polit & Beck 2017:870). Snowballing of participants was done until data saturation was achieved, which occurred with 14 participants. Data saturation denotes a stage in data collection when no new insights are identified and data begin to repeat, leading to redundance, signifying that an adequate sample size is reached (Hennink & Kaiser 2022:2). Taping from a systematic review by Bartholomew et al. (2021:6), 14 participants were deemed sufficient to answer the research question as the phenomenological approach of interpretative nature has a sample size of 11.61 on average, with a range of 5–50 participants, while descriptive or transcendental phenomenology studies has an average of 12.14 sample size, with a range of 1–31 participants.

Only nursing students who were in the second, third and fourth-year of study, who had confirmed COVID-19 positive test results, were considered to participate in the study, and this served as inclusion criteria. Nursing students at the first-year level were not included in the study because they had just commenced training in 2022 when COVID-19 cases declined.

### Data-collection procedures

A total of six face-to-face individual and eight telephonic interviews were conducted between June and August 2022. Participants gave signed consent prior to the commencement of the interviews. For face-to-face interviews, three participants agreed to meet the researcher at the campus in an empty classroom, while the other three were interviewed at the private site in the hospital garden. Both venues were safe, private and free from noise. To direct the interview process, an interview guide was used. The guide was developed by the researchers and consisted of two central questions, which was; ‘tell me about your experience of being in isolation after testing positive for the COVID-19 virus?’ This was followed by prompts that focused on understanding the source of resilience, the impact of isolation on human connectedness and how they managed symptoms while in isolation. Moreover, probing assisted researchers in understanding the lived experiences of the participants. The interview guide was pretested with two participants who were not part of the main study, and this was done to ensure credibility and confirmability of the study.

All interviews were conducted in English. An audio recorder on the researcher’s smartphone was used to record face-to-face interviews, while telephonic interviews were conducted on a recorded phone call, and then all were transcribed verbatim. Consent to record was also sought from participants before recording. The recording was needed to avoid loss of data and helped researchers in the data analysis process. In addition, field notes were taken to capture the researcher’s reflections and non-verbal cues during interviews. Considering that the current study followed a phenomenological design, the researchers kept research diaries whereby notes on reflexivity and bracketing of personal feelings and assumptions were made. The main points of the interview were verified with participants as a form of member checks, which also ensured study credibility. The duration of the interviews was 30–45 min and participants’ responses determined this duration.

### Data analysis

Simultaneously with data collection, researchers analysed data manually according to interpretative phenomenological analysis (Smith, Flowers & Larkin 2009:79). Researchers read through the transcripts several times individually to familiarise themselves with the data and gain new insights. They noted thoughts, similarities, differences and contradictions in participants’ responses on the page margins. They then transformed these notes into concise phrases, which represent codes. Phrases were listed, and then connections were made between them. While interpreting phrases, similar ones were grouped to form themes and subthemes. They were presented in a coding tree and then transferred into a table (Table 2). Notes taken as field notes were also considered during data analysis. The two researchers met in a face-to-face meeting to agree on final themes and subthemes. To ensure credibility and dependability, peer debriefing was conducted with one researcher who was not part of this project.

### Techniques to enhance trustworthiness

The four criteria of trustworthiness, which are credibility, dependability, confirmability and transferability, were followed in this study (Lincoln & Guba 1985:290–331). To ensure credibility, prolonged engagement was practiced by making sure adequate time was allocated for field work to ensure persistent observations and saturation of data. Data collection stretched from June 2022 to August 2022, with interviews lasting 30 min–45 min. Adequate time was necessary for building trust and rapport during recruitment and interviews with participants. Face-to-face interviews were audio recorded while telephonic interviews were conducted through recorded phone calls to further ensure credibility. For each interview, researchers shared transcripts and findings for confirmation of interpretations made. Furthermore, to ensure credibility, regular debriefing was conducted with peers who were not part of this research project.

Dependability was ensured by documenting of steps followed and decisions made during the project, as well as necessary documents such as interview transcripts and field notes. Additionally, researchers kept diaries for documenting personal reflections and notes to assist with bracketing personal assumptions and other related experiences. This was necessary, considering that the design followed is phenomenological. The researchers ensured research methods were described in detail to help other researchers make judgements on the transferability of current findings to their contexts. Confirmability was warranted by using extracts of quotes from participants to support each sub-theme in the findings.

### Ethical considerations

The Campus Nursing Research Ethics Committee at the University of Namibia has assessed the study protocol and granted ethical clearance (Ref SoN 49/2022) and a permission letter dated 12 May 2022. In addition, written informed consent was obtained from each participant prior to participation in the study. Confidentiality was maintained by conducting interviews in private venues with no audience and in the case of telephonic interviews, the researchers confirmed with participants that no other people listened to the conversations. All audio recordings and transcripts were stored in password-protected devices. Anonymity was ensured by using numbers instead of participants ‘names during interviews and in reporting of findings.

## Results

The study consisted of 14 participants, six were males and eight were females. Their ages ranged from 19 years to 25 years. Four students were in the second year level, five in the third-year and five in the fourth-year level of nursing training. Eight participants tested positive for the COVID-19 virus in 2020, while six tested in 2021, only one tested positive in 2020 and again in 2021. Eleven of the participants indicated they lived with three to 11 family members in their houses, whereas three lived alone in the university hostel and rented rooms. None of the participants was hospitalised during the COVID-19 treatment but eight were admitted into community-based isolation centres, while the other six were instructed to self-isolate at their places of residence. Table 1 shows the demographic information of participants.

**TABLE 1:** Demographic information.

Variables	Range	19–25 years
Gender	Males	6
	Females	8
Isolation status	Self-isolate at places of residence	6
	Community-based isolation centre	8
Study level	Second year	4
	Third-year	5
	Fourth-year	5

### Themes

Researchers read through the transcripts several times individually to familiarise themselves with the data and get new insights. They noted thoughts, similarities, differences and contradictions in participants’ responses on the page margins. They then transformed these notes into concise phrases, which represent codes. Phrases were listed and then connections were made between them. While interpreting phrases, similar ones were grouped to form themes and subthemes. They were presented in a coding tree and then transferred into a table (Table 2). Five main themes and associated subthemes were conceptualised from the data, which are presented in Table 2.

**TABLE 2:** Themes and subthemes conceptualised from data analysis.

Themes	Subthemes
*A physiological spectrum*	The onset of symptoms
	Symptoms experienced
	Managing symptoms while in isolation
*A psychosocial spectrum*	Anxiety disorders
	Sense of imminent death
	Living in limbo
	Fear of transmitting the virus
	Stigma and discrimination associated with COVID-19
*The value of isolation*	Being a role model
	Protection of loved ones
*Source of resilience and coping mechanisms*	Remaining optimistic
	Learning new skills
	Family support
*Impact of isolation*	Impact on academic life
	Impact on human connectedness

COVID-19, coronavirus disease 2019.

#### Theme 1: A physiological spectrum

**The onset of symptoms:** Participants indicated they had physical contact with other people who tested positive. They were placed in quarantine when symptoms began to appear and then transferred to the COVID-19 community-based isolation centre or self-isolated, while others showed symptoms without being a suspect or coming in contact with a person who had tested positive. Overall, they had very minor symptoms at the beginning, which later worsened and progressed into a serious illness as the days went by:

‘One day as I was coming from campus I was hit by a heavy wave of dizziness, I had a rapid test done, which came out positive and I was really shocked because as for me I was always taking the set precautions to avoid getting infected.’ (Participant 2,19 years old, Male, 3rd year student)

‘As for me the symptoms started developing three days after I had visited an aunt’s house for a weekend, I did not pay much attention to them as I thought it was just one of the ordinary common colds and I would get through it, until the fifth day when things drastically became worse where I felt like my soul was leaving my body then I went to see the doctor who then immediately did a COVID-19 PCR test then I was declared to be COVID-19 positive and I needed to be on antibiotics for at least five days.’(Participant 11, 22 years old, Female, 3rd year student)

**Symptoms experienced:** Common symptoms listed were feeling feverish, headache, fatigue, sore throat, dry cough, flu and body aches. Moreover, others complained of poor appetite, sudden weight loss, anosmia, aphonia, agues, vomiting, joint pain and dyspnoea:

‘I suffered from high fever and body pains that tortured every part of my bone and muscle I felt numb due to severe pain, a walk to the bathroom was like a marathon run.’ (Participant 6, 23 years old, Male, 4th year student)

‘I had a really bad cough, I coughed throughout the day and night, it is like my lungs have a sack of rice and water when I take a deep breath, I felt pressure in my chest.’ (Participant 3, 22 years old, Female, 3rd year student)

‘I lost my voice, sometimes I could not make any sound at all, I sounded like I frog.’ (Participant 10, 21 years old, Female, 2nd year student)

**Managing symptoms while in isolation:** Participants indicated that they received prescriptions of painkillers and antibiotics from their doctors and nurses who visit the isolation centre to help relieve symptoms. Moreover, they also bought extra supplies from the pharmacies and used some home remedies:

‘The doctor prescribed for me erythromycin, cefixime, amoxicillin, ascorbic acid, and multivitamins, I was also steaming with hot water and eating a lot of oranges.’ (Participant 6, 23 years old, Male, 4th year student)

‘I made sure I get enough bed rest so my body to properly recover, I kept myself warm and avoided cold beverages.’ (Participant 3, 22 years old, Female, 3rd year student)

‘I was taking immune boosters and did steaming with anaconda and scorpion cream.’ (Participant 4, 21 years old, Female, 3rd year student)

#### Theme 2: A psychosocial spectrum

**Anxiety disorders:** Participants expressed feelings of panic, being scared, lost hope and having moments of paranoia, and others were worried too much and lonely while in isolation:

‘A lot of bad thoughts occurred, I almost like I lost the willingness to live, my mental health was hit, I was like I lost all my goals, so I cried.’ (Participant 7, 20 years old, Female, 2nd year student)

‘It was too lonely in the isolation centre you keep on doing the same thing over and over same routine every day it was not nice at all.’ (Participant 14, 22 years old, Male, 2nd year student)

‘I was stopped from entering the premises of one of our neighbours where I used to go past time activities. They accused me of carrying the virus that would lead to their death, I was so distressed by this.’ (Participant 8, 25 years old, Female, 4th year student)

‘It was difficult because I was alone, especially on my birthday, I was isolated physically and mentally. A lot of bad thoughts were piling up in my head my mental health took a hit I lost the willingness to live, it was like I lost my goals so I cried.’ (Participant 10, 21 years old, Female, 2nd year student)

**A sense of imminent death:** Participants experienced the feeling that their own death is approaching. Reading about COVID-19 case mortality statistics made this feeling worse, especially after hearing of the death of young people or someone known to them:

‘All media platforms talked about how fatal the disease is. As death cases were increasing so did my stress levels especially when the youths were dying. I heard people younger than me succumbing to the illness, and that’s when I realised the disease is not here to joke with anyone, so thoughts of what if die started developing.’ (Participant 8, 25 years old, Female, 4th year student)

‘As the symptoms persisted and new ones started developing, I was scared, I was asking myself what if I go to sleep at night and not wake up in the morning, I felt like I might not be alive the next hour.’ (Participant 1, 23 years old, Female, 4th year student)

**Living in limbo:** There was confusion caused by information shared on social media, newspapers and from published articles online. This created a state of uncertainty and turmoil in nursing students’ lives. Doubts about the accuracy of the information provided by the media, being cluttered with information and exacerbated anxiety by hearing conflicting news led to confusion among victims:

‘I used to check the internet, unfortunately, almost all the information on the internet about the disease was full of suspicion and speculation. One website would write something and another website would write else and interestingly every TV channel would say something different about the disease. I really could not figure out which one is true.’ (Participant 9, 24 years old, Male, 3rd year student)

‘We received a lot of conflicting information about the disease every day for example one would take vitamin C, but another would say no, it is too acidic and would exacerbate coughs one would say get vaccinated and another would say it is not safe.’ (Participant 7, 20 years old, Female, 2nd year student)

**Fear of transmitting the virus to others:** Participants were concerned about infecting other people and worried about harming their family members especially children and elders because of being in high-risk groups, which created a sense of guilt in them:

‘I was afraid of transmitting the disease to my family members, especially my father who is hypertensive.’ (Participant 13, 20 years old, Male, 2nd year student)

‘I felt guilty because of the possible risk that my family would be exposed to catching the virus from me.’ (Participant 8, 25 years old, Female, 4th year student)

**Stigma and discrimination associated with COVID-19:** Participants experienced bad words, discriminatory comments and stigmatising treatment from their community and family members after being discharged from the isolation centre:

‘Even though I was declared clear from COVID-19 after 14 days of self-isolation, some members of my community still looked at me, in their minds, they still viewed me as a positive case even though I was fully recovered.’ (Participant 12, 24 years old, Female, 4th year student)

‘Discrimination against the virus is quite high, people still regard a patient as a threat or one who would still pose danger to them even after ten days of isolation.’ (Participant 3, 22 years old, Female, 3rd year student)

‘People in my location after discovering that I tested positive were no longer associating with me and this was even extending to my family members.’ (Participant 2,19 years old, Male, 3rd year student)

#### Theme 3: The value of isolation

**Being a role model:** Being nursing students, participants felt like agreeing to be in isolation after testing positive for COVID-19 made them exemplary in their community and expected everyone to follow while fighting to curb the pandemic:

‘I am a nurse and have to live by rules.’ (Participant 4, 21 years old, Female, 3rd year student)

‘I felt I did not have a choice, just have to go for isolation, the community is looking upon on us.’ (Participant 8, 25 years old, Female, 4th year student)

**Protecting loved ones:** Participants had a deep concern about becoming an agent of infecting others, especially when there is a person with underlying comorbidities such as diabetes, hypertension, who is susceptible to contract COVID-19. Therefore, they adhered to all isolation rules:

‘This isolation thing was not for me; it was to protect others.’ (Participant 7, 20 years old, Female, 2nd year student)

‘I did not want to go for isolation, but I stayed with my grandmother who suffers from diabetes.’ (Participant 8, 25 years old, Female, 4th year student)

#### Theme 4: Source of resilience and coping mechanisms

**Remaining optimistic:** Although isolation was lonely and stressful, participants believed that their lives were significant and purposeful. The determination to achieve their goals and a sense of fulfilment in life keep them strong. In addition, having hope of getting well provided them with a positive mentality needed to fight the COVID-19 virus:

‘It only requires a positive mindset; I believe I survived because I had hope that I will get well soon.’ (Participant 4, 21 years old, Female, 3rd year student)

‘What kept me going or motivated me was the fact that I am a third-year student I came this far let me not give up on myself I do not have much time left to complete my study, I made sure to remember my goals and focused on them.’ (Participant 8, 25 years old, Female, 4th year student)

**Learning new skills:** Interestingly, participants took the opportunity of being in isolation to catch up on the events they had been procrastinating, for instance, some learned new cooking and gardening skills as a coping strategy and source of resilience:

‘I tried to stay as active as possible so on days that I felt t my best I tried to transplant my flowers, try out new food recipes.’ (Participant 13, 20 years old, Male, 2nd year student)

‘I have attempted to do knitting while in isolation, I did knit and a lot of handwork practice in my primary school years and had completely forgotten but this time I just followed steps on YouTube.’ (Participant 1, 23 years old, Female, 4th year student)

**Family support:** As a source of resilience and coping mechanism, participants relied on strong support from their family members while battling with COVID-19 in isolation. Support was described in the forms of phone calls, sometimes getting food prepared by family members, motivating messages and Bible verses:

‘My family was very supportive in terms of checking up on me while in isolation. They called every day and asked if I needed anything and they would bring it as soon as possible they also called via WhatsApp video call shared jokes with me showed me around the house so I felt like I was at home.’ (Participant 4, 21 years old, Female, 3rd year student)

‘My people made sure I get everything I needed, they have really put all effort, you know… I was really feeling it that we were fighting the virus together.’ (Participant 12, 24 years old, Female, 4th year student)

#### Theme 5: Impact of isolation

**Impact on academic life:** Participants had fears that they were missing out on learning activities as they were not able to attend clinical practice, simulation and theory classes while in isolation:

‘My fear was that maybe the lecturers and clinical instructors will not be available to demonstrate for me the missed out learning demonstration so how will I be able to catch up on my own?.’ (Participant 9, 24 years old, Male, 3rd year student)

‘Since I had to be in isolation, I missed out on all practical activities that happened within those fourteen days, but theoretical wise I was up to date because I had every good friend of mine that would send me recorded audios of the lesson which I listened to because it was really hard to make notes on my own from all the hand-outs and slides.’ (Participant 11, 22 years old, Female, 3rd year student)

‘I stayed away from practical and school for 14 days, a lot was going on in my head I was overthinking everything, what if I do not pass, what if the lecturers do not give me extra time to complete my books and assessments but by God’s grace it all went all I have the most understanding lecturers ever.’ (Participant 3, 22 years old, Female, 3rd year student)

**Impact on human connectedness:** Participants felt that their social life was negatively affected by being in isolation. They expressed how they missed human closeness, such as hugging, handshakes, laughing together and being in the physical company of someone. They have indicated that talking via phone is not comparable to face-to-face communication, there is more value and closeness attached to being in the company of other people:

‘I missed the connection with other people while in isolation, talking to someone over the phone is not enough. Like me I was in a single room, the caretakers came in one second and left immediately due to precaution measures.’ (Participant 14, 22 years old, Male, 2nd year student)

‘It is true when they say we are social beings… it is not good to be alone day and night.’ (Participant 2, 19 years old, Male, 3rd year student)

‘We strictly used to be in-door and not interacting with one another and no visitors. If visitors come, you do not see them, they stop at the main entrance of the center. Like our relatives were more encouraged to make telephone inquiries than come to see us. Another issue … If you need the assistance of another person, you ask in a discreet manner and be careful for other people not to notice, they must not see that you interact with other people.’ (Participant 11, 22 years old, Female, 3rd year student)

Moreover, participants expressed that because of isolation, it was not possible for family members or anyone from the community to care for them. This was again their cultural practice; during any illness, the sick person is cared for by who are close to them, either family members or people who reside in the same community:

‘When we are sick, we are cared for by our people, they remain near, help with feeding, personal care, and also talking about the illness. This was not possible during isolation.’ (Participant 4, 21 years old, Female, 3rd year student)

## Discussion

This study explored how nursing students who tested positive for the COVID-19 virus had experienced the process of being in isolation and understanding their source of resilience. Findings indicated physiological and psychological spectrums, the value of isolation, the source of resilience and coping mechanisms and academic isolation. As with other patients who tested positive for the COVID-19 virus, the current study participants experienced symptoms that were of acute onset and got worse with days, while some remained asymptomatic. Although some patients remained asymptomatic with positive COVID-19 results, their isolation remained important because of the high contagiousness of the virus (Mercan, Digin & Bulut 2021:122). Centers for Disease Control (CDC 2024:n.p.) reported that symptoms of COVID-19 start 2–14 days after exposure. Symptoms usually start as mild and progress to severe illness. The common 10 self-reported symptoms of COVID-19 are fever, headache, anosmia, ageusia, fatigue, cough, sore throat, dyspnoea, diarrhoea and runny nose (Alanazi et al. 2020:5). Those were similar symptoms reported in the current study, which were managed following nurses and medical doctors’ prescriptions as well as home remedies while in isolation.

For psychological reactions while in isolation, the findings of the current study are similar to a Bangladeshis study that indicated that patients had sleepless nights, loneliness, frightened and distressed feelings and fear of death (Fahmi et al. 2021:46). Other authors reported on patients’ nervousness, fear, worries, uncertainties and lack of confidence in treatment, loss of motivation, meaning of self-worth while in isolation (Pei et al. 2021:1115; Williams et al. 2020:3). While nursing students were reported to show depressed moods and decreased motivation because of lack of socialising with friends, boredom and seeing lots of negative news on COVID-19 (Laczko et al. 2022:394). It is worth noting that despite a psychological spectrum of symptoms experienced by nursing students while in isolation, no participant mentioned seeking professional help regarding this, except for physical or physiological symptoms. This is contrary to Kerbage et al. (2021:1412), who reported that nursing students got professional help from counsellors and psychiatrists to help them cope mentally.

Human beings are a social species, and it is in their nature to interact with others. Therefore, social interaction and relationships with others are significant for the mental well-being of people in all age groups (Filho et al. 2021:18). However, in the current study, nursing students valued isolation over social and physical interaction for the purpose of serving as role models and protecting loved ones from the COVID-19 virus. This indicates that nursing students display professional identity and see themselves as valuable members of the community who have others looking upon them. At the same time, students display caring attitudes towards their family members by accepting wholeheartedly to be in isolation to protect them. Correspondingly, a previous study confirmed that students with high perceived stress were those most likely to have a COVID-19 diagnosis in their household (Laranjeira et al. 2021:6).

Factors such as an individual’s personality, psychological support, duration of isolation, group dynamics and coping strategies or mechanisms are linked to the development of complications associated with isolation and confinement (Choukér & Stahn 2020:2). To prevent complications of being in isolation and attain a great level of resilience, participants in the current study remained optimistic, learned new skills and depended on their family support as a source of resilience and coping mechanisms. Similarly, a study conducted on nursing students in Malawi reported that participants were optimistic and hoping for recovery, just like other community members who tested positive and recovered from the virus (Baluwa et al. 2021:1393). Moreover, common coping strategies of students included acceptance, planning and seeking emotional support (Babicka-Wirkus et al. 2021:7). Similarly, Liozidou et al. (2023:1268) reported acceptance, positive reframing, planning, active coping and self-distraction through work or other activities as patients’ coping mechanisms during the COVID-19 pandemic isolation. On the same note, healthcare workers cope and become resilient by accepting the situation. Nevertheless, they resort to religious activities, emotional coping through self-encouragement, seeking counselling from colleagues, reading about new developments on the disease and bulk purchasing of basic necessities (Taremwa et al. 2023:555). A study that reported on nursing students’ resilience during the COVID-19 pandemic indicated that students remained positive, stayed connected with others and practiced self-help activities such as yoga, listening to music and mindful medication (Kerbage et al. 2021:1412).

The COVID-19 pandemic had a negative impact on nursing education. This is because there has been a disruption in the way nursing students are socialised. Usually, students learn together during face-to-face encounters, sit together on campus, work in groups and meet up with their lecturers (Agu et al. 2021:155). These experiences were abruptly replaced by virtual learning. Nursing students missed the social dimension of learning, had concerns related to not reaching learning outcomes and challenges of clinical placement during the pandemic (Rohde et al. 2022:7). Moreover, teachers were not supportive and stress related to the pandemic had an impact on students’ grades (Laczko et al. 2022:395). In the current study, participants had concerns related to missing out on learning activities because of prolonged absenteeism while in isolation. During the time they were in isolation, others were able to attend clinical practice, simulation and theory sessions.

Another impact of isolation reported in the current study is on human connectedness. The current study resonates with Kerbage et al. (2021:1411) who reported that during the COVID-19 pandemic, nursing students experienced being not connected to others because they did not have friends and mostly stayed indoors. In addition, Smallen (2021:2893) reported that there were struggles with meaningful connections with others. Overly, lack of connectedness with others was reported to be a major social and health problem of people in all age groups during the COVID-19 pandemic (Jacobs & Ellis 2021:6). This has negative consequences considering the fact that in Africa, wellness and caring for sick people are rooted in the community (Chigangaidze et al. 2022:322). Similarly, according to the cultural philosophy of participants and the setting where the current study was conducted, a sick person is cared for by family members and to some extent the community at large. Family and community members support a sick person through physical care and psychosocial support to help them cope with the burden of diseases and accompanying challenges. Decisions related to care practices are taken in full consultations with others and are not an individual endeavour. Henceforth, the community develops resilience together through the spirit of *ubuntu* philosophy, which is significant to maintain mental health during stressful situations such as the COVID-19 pandemic.

The findings have implications for nursing education and practice, in preparation for future pandemics, specifically in promoting the resilience and mental health of students. Considering the fact that the COVID-19 pandemic had severe psychological effects on nursing students in isolation, it is of great importance to establish a coordinated counselling and psychological support system. In addition, educators should come up with remedial activities to assist students who missed out on learning opportunities while in isolation. Alternatively, to keep students engaged with learning, educators should design virtual learning activities and work closely with family members to conduct regular virtual visits of students while in isolation. Henceforward, student counsellors should keep a register of students who have tested COVID-19 positive and undergone isolation; this is for monitoring, identifying and management of post-traumatic stress disorders. Considering that individual strengths and the support of family members were central to students’ coping and resilience while in isolation, future researchers may explore best practices for self-care measures during isolation and how human connectedness may be maintained during isolation practices. Moreover, researchers may develop strategies for family members to support people in isolation during pandemics.

### Strength and limitations

The strength of this study lies in the fact that empirical data were collected from nursing students as patients in isolation, adding to the scanty literature available on experiences of nursing students who tested positive for COVID-19 and how they survived the process of being in isolation. As a mode of data collection, both telephonic and face-to-face interviews were utilised. Telephonic interview is restricted in observations of non-verbal communication cues that may be used to display emotions, thus limiting researchers’ ability to identify emotions. To overcome this limitation, the researchers conducted interviews during the time suitable for participants to ensure less interruptions to enable them to reflect on their experiences. In addition, adequate time was allocated for interview to allow for researcher to explore and understand responses, which also include interpretation of emotions. Moreover, this study required participants to reflect on their past experiences; therefore, its retrospective nature revived bad memories. This was seen as a limitation in this study; therefore, an option for referral to the social worker for counselling was made available to all study participants. Considering that participants reflected on past experiences, recall bias could be a possibility in the study. Therefore, during data collection, the researchers engage in deep exploration and probing questions to enhance participants to recall experiences. Prolonged engagement and conducting data collection till saturation is reached allowed for participants to recall experiences and for rich data to be collected.

## Conclusion

It is concluded that nursing students who tested positive for the COVID-19 virus had experienced isolation as a period of reflecting on how their symptoms commenced and how they progressed from being minor to major symptoms. Moreover, they had undergone a psychosocial spectrum. Isolation was expressed to be valuable as it made students role models who protect their loved ones from the COVID-19 virus. As a source of resilience and coping mechanisms, students remained opportunistic, learned new skills and leaned on family support. Despite that, the process of being in isolation had an impact on academic life and human connectedness. The findings have practical implications for nursing education and practice, in preparation for future pandemics, specifically in promoting resilience and mental health in students.
